# Empirical Confirmation of Creative Destruction from World Trade Data

**DOI:** 10.1371/journal.pone.0038924

**Published:** 2012-06-18

**Authors:** Peter Klimek, Ricardo Hausmann, Stefan Thurner

**Affiliations:** 1 Section for Science of Complex Systems, Medical University of Vienna, Vienna, Austria; 2 Center for International Development and Harvard Kennedy School, Harvard University, Cambridge, Massachusetts, United States of America; 3 Santa Fe Institute, Santa Fe, New Mexico, United States of America; 4 International Institute for Applied Systems Analysis, Laxenburg, Austria; University of Zaragoza, Spain

## Abstract

We show that world trade network datasets contain empirical evidence that the dynamics of innovation in the world economy indeed follows the concept of *creative destruction*, as proposed by J.A. Schumpeter more than half a century ago. National economies can be viewed as complex, evolving systems, driven by a stream of appearance and disappearance of goods and services. Products appear in bursts of creative cascades. We find that products systematically tend to co-appear, and that product appearances lead to massive disappearance events of existing products in the following years. The opposite–disappearances followed by periods of appearances–is not observed. This is an empirical validation of the dominance of cascading competitive replacement events on the scale of national economies, i.e., creative destruction. We find a tendency that more complex products drive out less complex ones, i.e., progress has a direction. Finally we show that the growth trajectory of a country’s product output diversity can be understood by a recently proposed evolutionary model of Schumpeterian economic dynamics.

## Introduction

Joseph A. Schumpeter held that the key mechanism of economic development is radical innovation [1], [2]. In his view the Walrasian economic equilibrium is continuously disturbed by actions of entrepreneurs, introducing novel goods and services in the market. These innovations may replace existing goods and services and thereby impact related industries. If this happens as a cascading process it is called *creative destruction*. Current colloquial examples of how once market-dominating companies lose their position due to creative destruction include instant photography or printed newspapers in light of the age of digitalization. But is this only the driving force behind major shifts in industrial production and long-term business cycles, or does it also condition economic change on much shorter time-scales?

This question has been addressed using firm entry and exit dates [3], job creation and destruction rates [4], within specific countries [5] or within specific industries [6]. These works capture the complex interplay between factor re-allocation and productivity growth in existing economic sectors. However, little is added to our understanding of the impact of emerging industries on the development of already established parts of the national economy. If a new industrial branch emerges, how does this impact other economic sectors?

Traces of the creation of unprecedented industries can be observed using world trade data. We study the dynamics of the diversity of export products and show that the process through which it changes follows the patterns of creative destruction. The set of products a given country exports reveals the presence of the nontradable inputs or capabilities that the products require for their production (e.g. specific productive knowledge, infrastructure, legal system, labor skills, regulations, etc.) [7], [8]. In this view capabilities are elementary building blocks and each product requires a combination of them to be manufactured. A change in the diversity of a country’s product basket indicates a change in its set of capabilities. New capabilities may lead to new products and the abandonment or substitution of already existing products or capabilities. Products requiring one of these abandoned capabilities as input will then disappear, while simple products that require only subsets of capabilities of more complex products may disappear as they are unable to compete for these inputs – creative destruction at work.

In this article we show that typically within a country clusters of products appear simultaneously in bursts. We interpret such a burst as the acquisition of a novel capability which is required as input for each of the newly appearing products. In the years following creative bursts there is an increased chance that already existing products will cease to be exported, that is, emerging industries effectively push out the old ones. The novel products tend to be more sophisticated and complex than the disappearing products, i.e. progress has a direction. Interestingly, we do not observe a higher probability of product appearances following disappearance events of products (see Fig. S2). This means that the canonical mechanism of filling “market niches”, if existing, does not operate in the mode of re-populating previously abandoned market niches. We confirm empirically that creative destruction, cascades of competitive replacement, plays an important role in the development of national economies. We base our results on the World Trade Flows database compiled by the National Bureau for Economic Research [9].

We apply a recently proposed Schumpeterian diversity dynamics model [2] to account for the evolution of the product diversity of countries. In this model entrepreneurs use and combine available capabilities to create novel goods and services and substitute them for already existing ones. The result is a model economy in a self-organized critical state, characterized by creative and destructive co-evolutionary avalanches. The changes in product diversity and the distribution of product appearances and disappearances observed in world trade data are well explained by this model. The process of creative destruction has previously been modeled within the framework of endogenous growth theory [10]–[12] in works focusing on endogenous technological change [13]–[15]. These models are typically build around the same production functions used in neoclassical growth models (e.g. Cobb-Douglas). Instead of modeling technological advance through an exogenous growth rate, often an additional R&D sector is introduced, developing patents and selling them to production firms which become monopolists, leading to an imperfect market. The Schumpeterian diversity dynamics model [2] used here is completely detached from these developments.

## Materials and Methods

### Diversity Dynamics

The World Trade Flows database [9] contains exports of approximately 200 countries over the years 1984–2000 in about 800 product categories (4-digit SITC rev.2 classification). We include only those countries in the analysis which have a population of at least 1.2 million people and total exports of at least 1 billion USD, for more information see Text S1. We label the countries contained in this database by *c*, the product category by *p* and the year by *t*. The export values 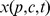, denominated in USD are then extracted. As a diversification measure of country *c* in year *t* the number of products with nonzero export values is used, that is, 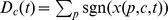. The total number of countries is denoted by 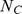, the number of products by 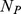.

**Figure 1:**
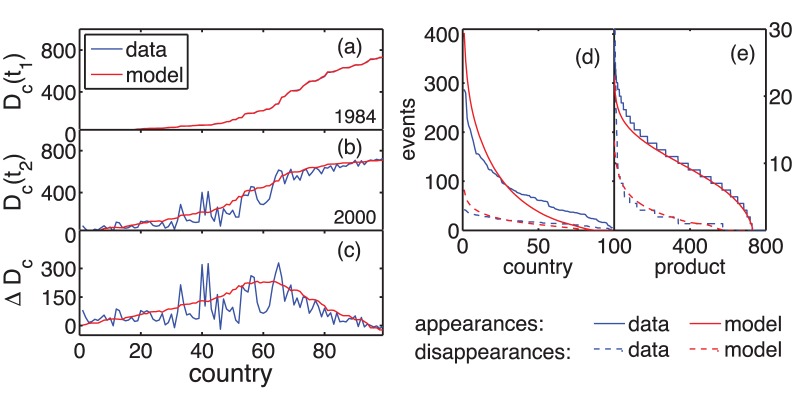
Development of export product diversities. Countries’ export product diversities for (a) 1984, (b) 2000 and the net change in diversity between these years (c) are shown for world trade data (blue lines) and the Schumpeterian diversity dynamics model (red). There is a general tendency towards increased diversity which is strongest for countries having initially an intermediate product diversity. The results for diversity change are obtained by using only the subset of countries having non-zero export values in each year. The number of (dis)appearance events for each (d) country and (e) product are shown in the panel to the right for data and model. The model does not only reproduce the general trend of increasing diversity, but also the detailed profile of how many appearance and disappearance events could be observed for different countries and products. Note that in (a)-(c) countries are ranked by their initial diversity, whereas in (d) they are ranked by their number of events. For a goodness-of-fit test between model and data we report the correlation coefficient *p* between data and model results, and the *p*-value up to which the null hypothesis of no correlation between model and data can be rejected. For the final diversity in (b) we find 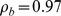, 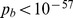 and for diversity changes in (c) 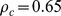, 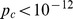. For the appearances (disappearances) per country in (d) we find 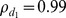, 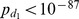 (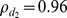, 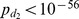), for appearances (disappearances) per product (e) 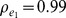, 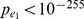 (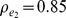, 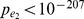).

The change of diversity of product exports in world trade data over a timespan of 16 years is shown in Fig.1(a)-(c). For each country index *c* the diversity 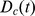 is shown for two different years, 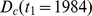 in Fig.1(a) and 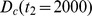 in Fig.1(b). The net change in diversity for each country between 1984 and 2000, 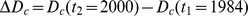 is shown in Fig.1(c). Values from trade data are shown in blue and are compared to results of the Schumpeterian diversity dynamics model (in red, to be explained below). There is a general trend towards increased diversity. Countries with a relatively low or high diversity in 1984 show smaller fluctuations in diversity than countries with intermediate initial diversity. That is, countries with a low diversity tend to stay poorly diversified, fully diversified countries stay fully diversified. In between there is a regime of transition countries, some of them showing explosive growth in terms of their economic diversity. We show later that these observations can be described through the onset of a ‘creative phase transition’ [2], a distinguishing property of Schumpeterian diversity dynamics. Let us now empirically investigate the process by which the countries’ product export diversities change.

Let 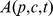 be a product indicator function for the appearance of product *p* in country *c* between year 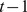 and *t*,
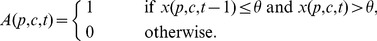
(1)Similarly the indicator function for a product disappearance is
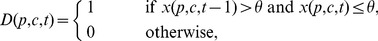
(2)with a threshold value set to 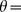 100,000 USD. For more details see Fig. S1. Fig.1(d) and (e) show the distribution of (dis)appearance events A (D) per country c and product p for world trade data and the Schumpeterian diversity dynamics model. They are far from being homogeneously distributed. For example, the number of appearance events per country varies between more than 300 and almost zero across different countries. Further there is a substantial number of products appearing in, say, five countries or less, while others appear in almost one third of all the countries studied. The model captures the functional form of these distributions. The number of appearances clearly exceeds the number of disappearances, consistent with the general trend towards higher diversity.

### Co-occurrence Analysis

Creative destruction can be described as a process started by introducing novel goods or services in a national economy. If successful, this stimulates the market introduction of related goods, complimentary to the newly introduced ones. Thereby a novel cluster of inter-related products can form. This cluster may render existing parts of the national economy obsolete. If this process is actually at work within national economies, one would expect two empirical facts to hold. (i) Products enter the market in creative bursts, i.e. not incrementally and (ii) the appearance of goods tends to foster the disappearance of other products. We quantify this with two different product indicators referred to as ‘Schumpeterian Product Indicators’ (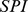). The first is introduced to assess co-occurrences of product (dis)appearances and is denoted 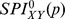. Here 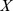 and 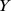 stand for any combination of events 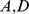 for product 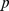. The second 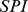 measures if an appearance or disappearance event of products at 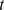 is correlated to an event of another product within the following 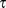 years and is denoted by 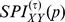.

The *empirical* marginal appearance (disappearance) frequency 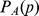 (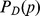) for each product is given by
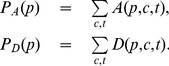
(3)The empirical number of co-appearances 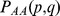 of products 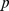 and 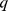 across all countries and times is,
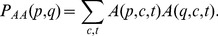
(4)The co-occurrence statistics 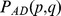 and 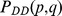 are obtained by appropriately substituting disappearance events 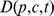 for 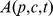 in Eq. 4 (by co-occurrence we will refer to any of the four possible pairs of events for two products p and q, that is (dis)appearance in p together with (dis)appearance in q). Obviously products with a relatively high number of appearances will also be more likely to co-appear. Consequently whenever one compares co-occurrence statistics of two different pairs of products one has to correct for this bias. A simple way to do this is to compare the number of measured co-occurrences of two products to their marginal (dis)appearance frequencies given in Eq. 3. For this we define the pairwise conditional co-occurrence measure as 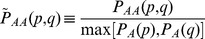. The idea is that given two products p and q we take the product with the higher appearance probability, say p, and measure how often q appears conditional on an appearance event of p in the same country. To quantify in how many co-occurrence events a given product participates, one can define an 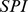 for simultaneous appearances 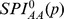 by
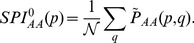
(5)where 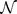 is a normalization factor given by 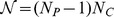 guaranteeing that the index lies within the range 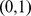 and is thus comparable across datasets of different sizes. It is straight forward to define 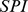
s for other pairs of events 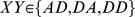.

We proceed to quantify to what extent an appearance event in year *t* is related to disappearance events at a later time 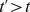. Assume a maximal lag of 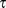 years between the two events, 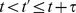. If not denoted otherwise, we work with a value of 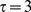 years. A simple measure is the number of all appearances of *p* which are followed by disappearances of *q* within the next 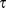 years in the same country and summed over all countries, i.e. 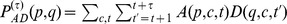. This number can be compared to the count of appearances of product *q* followed by disappearances of *p*, i.e. we exchange the roles of *p* and *q*. If there an asymmetry emerges when exchanging *p* and *q* in 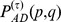, this indicates that *p* appearing before *q* disappears is observed more often than the other way around. In this spirit, for each product *p* a time-lagged 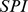 is defined as
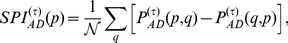
(6)This index is within the range 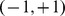. Intuitively, if one thinks of the appearance of *p* followed by disappearance of *q* within a country as a replacement, a positive value of 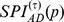 means that, on average, *p* replaces more often any other product *q* than *p* itself is replaced by *q*. The higher the 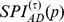 value for *p*, the higher the tendency that *p* can act as a substitute for other products.

For the purpose of statistical analysis we construct a surrogate dataset with the aim to destroy the correlations in the timing of (dis)appearances while the event statistics (as shown in Fig.1 (d) and (e)) are preserved. The surrogate data is prepared as follows. Each event is given by a triplet 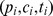 where index *i* runs over all appearance (disappearance) events in the data. For each *i* we fix 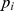 and 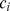 while shuffling the years 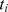 between the events in the triplets. Formally this defines a random permutation 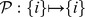 over the set of all event indexes 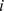. One may then calculate the co-occurrence measures from the triplet 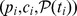 and average the result over many realizations of 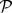. This ensures that the marginal distributions of the events (Fig. 1 (d) and (e)) remain unchanged while all correlations in the timing of appearances or disappearances are destroyed.

## Results

### Products Appear in Bursts

**Figure 2:**
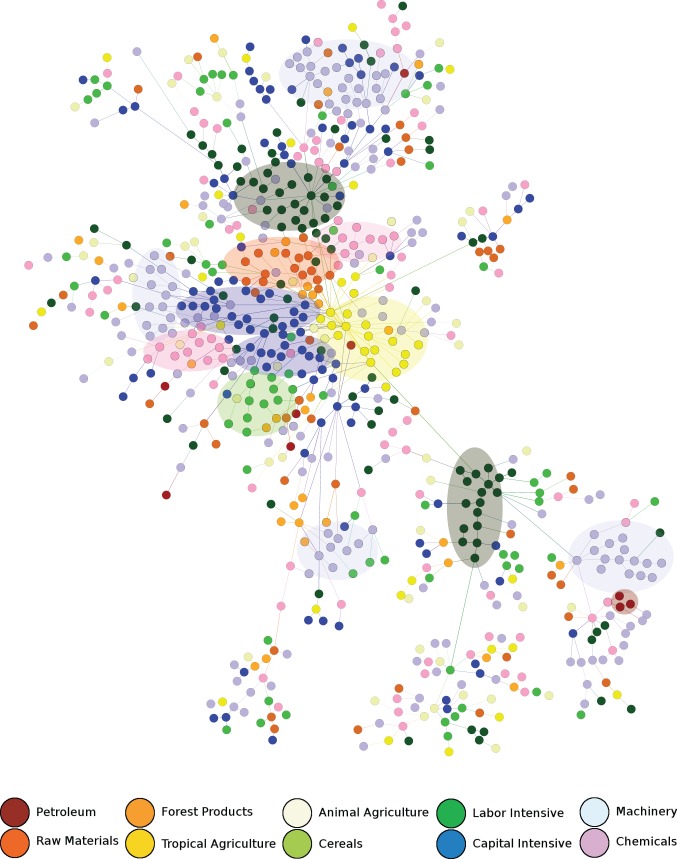
Maximum spanning tree for the network given by the co-occurrence measure 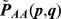. Some of the clusters of co-appearing products are highlighted as guides to the eye. It is suggested that these clusters of products require a common capability before they can be exported. Once a country acquires all of these capabilities, a creative burst of novel products which require them as input may be the consequence.

In Fig. 2 we show the maximum spanning tree for products obtained from the pairwise conditional co-appearance measure, 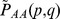 This allows to construct similarity matrices for product categories from which the maximum spanning tree, shown in Fig. 2, is constructed (see Text S3). Nodes in the network represent product categories according to the SITC rev.2 4-digit classification. The color of the nodes represents the products’ Leamer classes [16]. This is a classification based on relative factor intensities such as amount of capital, labor, land, skills etc. A similar visualization route has been followed to construct the product space [17]. Fig. 2 suggests that the clusters of co-appearing products require one or several common capabilities. Once a country acquires or upgrades these capabilities, it starts to report exports in these product categories. Their appearances are simultaneously observed as a creative burst within the same Leamer class. Some of these clusters are highlighted in Fig. 2 and point at a non-random structure in product co-appearance dynamics.

**Figure 3:**
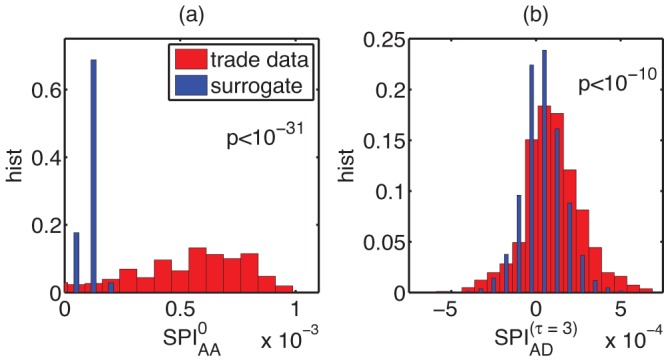
Histograms for Schumpeterian Product Index (a) 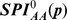 for co-appearances 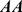, and (b) 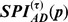 for 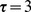 years. 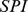
s are shown for the surrogate data (blue) and the trade data (red). The distributions for the trade data are significantly shifted to the right when compared to the surrogate data. The distribution in (a) suggests that products appear in bursts, while (b) implies that appearing products tend to drive other products from the market.

To make this structure explicit we compare 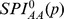 values for trade and the surrogate data, see histogram in Fig. 3(a). A huge difference between trade and surrogate data is found. This indicates the presence of strong temporal correlations between individual product appearances. Products appear simultaneously in bursts. A way to interpret this is that the co-appearing products require a common capability to be manufactured. When a national economy acquires this capability the corresponding cluster of products can appear. In this sense the observation of a newly emerging cluster of inter-related products serves as a proxy for a country’s development of a novel capability. This process is not unique within a given country, it can be observed in a substantial fraction of them (since the indicator values are averages calculated over more than one hundred countries). This may hint at common patterns in the diversification trajectory of individual national economies.

### Creative Destruction at Work

There is a tendency for specific products to substitute or replace others. This can be seen as deviations in the distribution of indicator values 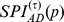 between world trade and surrogate data, see Fig. 3(b). A high value of 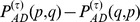 corresponds to the dominance of the pattern: ‘product 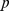 appears and 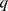 disappears later in the same country’ in a large number of them independently. This is a direct fingerprint of creative destruction at work, i.e. emerging industries pushing out the old. It is interesting to note that the opposite process – the disappearance of a product is followed by the appearance of another one – is not observed to a significant extent, see Figs. S2 and S9. For the 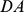 case surrogate and data 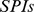 are practically identical. To measure the statistical significance of deviations from the surrogate data we formulate the null hypothesis that both surrogate and trade data are drawn from a normal distribution with the same mean. The 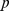-value using the alternative hypothesis that the trade data shows a higher mean than the surrogate data is computed for all four possible combinations: 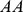, 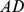, 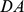, and 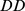. The results are listed in Table 1, together with 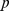-values from various robustness tests as described in Text S2. This series of tests confirms that the results above are not a consequence of trivial effects such as fluctuations in the trade records (case ‘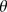 = 200 k USD’), the artificial creation of product categories due to changes in reporting schemes (‘all products with positive exports’) or the transition out of communism of former Soviet countries (‘Excl. FSU’), see Text S2. Results are also robust with respect to changes in the time-period (‘1989–2000’), the choice of the maximal lag 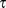 (‘
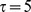
’,’
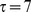
’) or the underlying dataset (‘UN Comtrade data’, [18]).

**Table 1:** 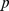
-values for 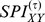 histograms from trade vs. surrogate data.

	*τ* = 3	*θ* = 200 k USD	all products with positive exports	cleaned data 1989–2000	Excl. FSU	*τ* = 5	*τ* = 7	UN ComTrade data
AA	0.98	1.00	0.93	0.96	1.00	0.94	0.84	1.00
DD	0.95	0.89	0.77	1.00	0.94	0.93	0.99	1.00
AD	<10^−10^	<10^−6^	0.0072	<10^−7^	<10^−5^	<10^−7^	<10^−3^	<10^−3^
DA	0.02	0.11	0.53	0.01	0.40	0.08	0.12	0.081

The column ‘
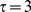
’ lists the results as described in the main text. In addition, robustness tests where conducted with results listed in separate columns. ‘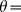  = 200 k USD’ uses the same set of countries and a threshold of 200.000 USD below which trade flows are ignored. In ‘all products with positive exports’ all products are included which have positive world exports in each year of the analysis. The column ‘1989–2000’ decreases the number of years included in analysis. Results excluding the FSU and CEE are listed in ‘Excl. FSU’. The maximal lag 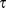 is then increased to 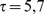. The last column reports results using the UN ComTrade dataset, as described in Text S1.

### Progress has a Direction

Is it true that more complex products replace less complex ones? Do products produced by richer countries replace products associated with poorer ones? To elucidate this, we quantify the change in complexity and income levels associated with individual 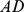 processes via a set of product indicators.

#### *Product income indicator PRODY*

This indicator for a given product is the weighted average of the per capita GDPs of countries exporting it. It is a weighted average of the income per capita of the countries that have revealed comparative advantage in that product [19]. A more sophisticated product, in principle, should be made by richer countries. Let us define the difference in 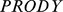 for products 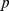 and 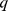 as 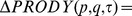 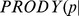 appears at 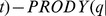 disappears at 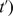 with 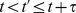.

#### *Product complexity indicato*r

The Product Complexity Index 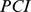 is an indicator for the economic complexity involved in manufacturing a given product [20]. The 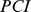 is a combination of the ubiquity of a product (i.e. the number of countries that make it) and the economic diversity of its exporting countries, see Fig. S3 for more details. Products with high 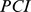 typically are made by few highly diversified countries which is indicative of high economic complexity [7]. The change in complexity 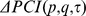 between two products is given by 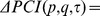 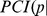 appears at 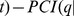 disappears at 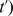, 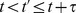. A comparison between 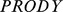 and 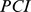 values for each country is shown in Fig. S3.

#### *Killer index*

We refer to 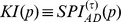 as the ‘Killer Index’ for each product 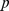. It measures the likelihood that the appearance of a given product 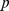 is observed with the disappearance of any other product in the next 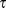 years.

#### *Extinction index*

The likelihood that a product disappears together with the appearance of any other product 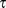 years earlier is expressed by its ‘Extinction Index’ 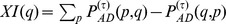. We set 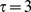.

**Figure 4:**
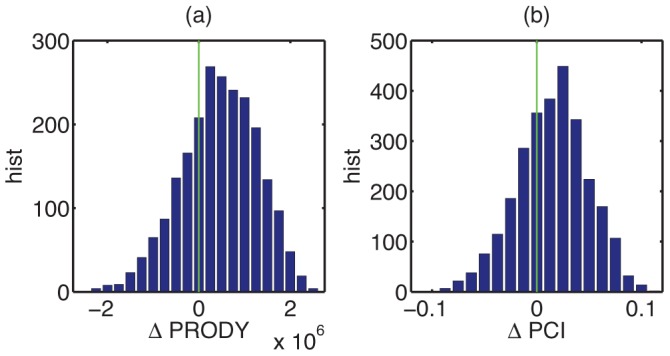
Histograms for change in (a) income level 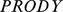 and (b) product complexity 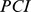 associated with individual creative destruction processes. There is a skew to the positive side in both of them, indicating that national economies are typically restructured into the direction of more complex and higher income products.

The creative destruction dynamics is dominated by products with a high value of 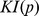 or 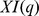. 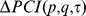 and 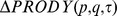 are thus measured for each 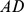 process with 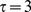, where the appearing (disappearing) product has one of the hundred highest values of 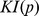 (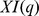). A positive 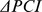 indicates an increase in economic complexity of the country where this 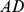 process was observed. A positive 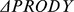 indicates that the country is upgrading its exports towards those made by richer countries. Histograms for 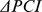 and 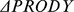 are shown in Fig. 4. There is a clear skew to the positive side for both quantities. Products with a higher tendency to be pushed from the market have in general lower economic complexity than products from appearing industrial branches.

One could imagine that this effect is stronger in emerging economies than in mature ones. To test this, one can compare 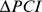 and 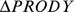 distributions for different economies by aggregating the countries into seven regions (Latin America and the Carribean, East-Asia and the Pacific, Middle-east and North-Africa, Sub-Saharan Africa, South-Asia, Western Europe and Northern America, Eastern Europe), as done in Figs. S4 and S5. A clear development towards higher economic complexity can be seen for Latin America, Eastern Europe and East Asia & Pacific. The other regions do not display such a strong trend. Western Europe and Northern America are already almost fully diversified and stay that way, whereas e.g. Sub-Saharan Africa stays in the low diversity regime. These results are discussed in more detail in Figs. S6, S7 and S8.

### Which Products are the Killers?

**Figure 5:**
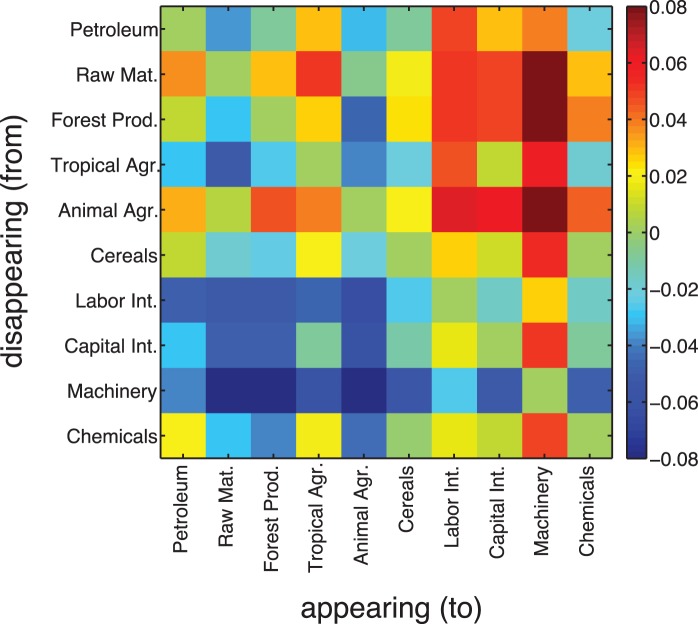
Transition matrix 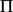 between Leamer classes. For each pair of classes 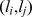 it is measured how often an appearance event of a product from class 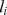 is observed followed by a disappearance of a product from class 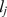, compared to the opposite direction. Rows are indexed by the appearing Leamer classes 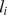, columns by disappearing ones 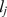. A positive value indicates an excess in creative destruction processes between the two classes (from row index to column index). The matrix is by construction anti-symmetric. There is a clear tendency of appearing labor and capital intensive products, as well as machinery and chemicals. Cereals or agricultural products tend to disappear.

Let us get a clearer picture about the products driving the creative destruction process. Are there Leamer classes whose products appear more often than others? Which types of products disappear instead? We consider the matrix 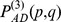. Each product can be assigned to one of ten Leamer classes, labeled by 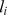, 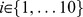. One can obtain a measure for 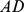 processes where products from class 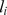 appear and products from 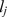 disappear by computing the mean value of 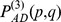 over all products from the respective Leamer classes. To this end 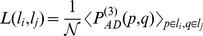 is defined. As before, we are only interested in the ‘net flow’ of 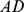 processes, the antisymmetric Leamer transition matrix, 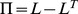. This is basically the same measurement strategy as discussed for 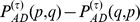, however aggregated to the level of Leamer classes. The matrix is shown in Fig. 5.

A positive value of 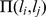 indicates that an appearance event of a product from class 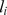 followed by disappearance of a product from 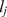, is more often observed than an appearance from 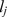 followed by disappearance in 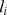. Given that 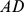 processes convey information about how national economies are re-structured over time, positive values in 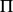 indicate which parts of the economy are abandoned or disappear because of which other parts. There is a clear trend. Capital and labor intensive products, as well as machinery and chemicals appear much more often than they disappear. Agricultural products and cereals tend to disappear. This intuitively confirms that markets re-structure themselves towards higher economic complexity, as measured by the 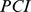 and observed in Fig. 4. Note that these observations can not be described by diffusion or migration processes of e.g. production facilities or capabilities from one country to another. Each measurement of appearances followed by disappearances takes place within a single country. A product appears within a country and another product disappears later within the same country.

### A Simple Model

**Figure 6:**
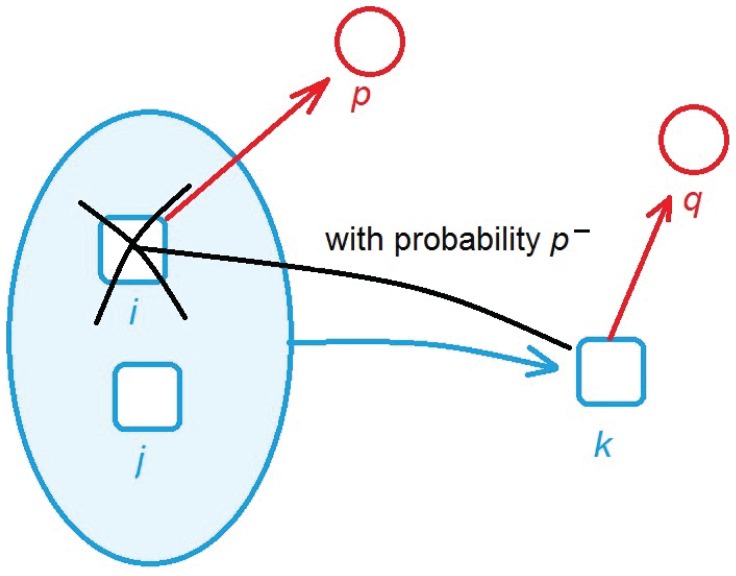
Illustration of the Schumpeterian diversity dynamics model. Capabilities are represented by blue squares, products by red circles. A country can acquire capability 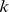 by combining other capabilities 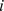 and 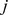. Each product requires a set of capabilities to be produced, e.g. product 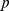 requires 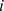 and 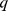 requires 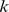. There is a chance that a novel product 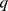 may act as a substitute for a product 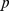 which is made from a subset of capabilities required for 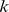. In this case 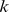 may effectively ‘destroy’ its preceding capability 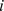.

A recently introduced Schumpeterian diversity dynamics model [2] centers around the assumption that countries have an evolving set of capabilities which firms combine to manufacture products, see Text S5. The economic state of a country is represented by two (high-dimensional) binary vectors, one indicating whether a country has a given capability, the other indicating whether a country exports a given product. These vectors evolve over time in each country. Capabilities can be acquired through entrepreneurs who combine existing capabilities. For example, an existing production infrastructure and a certain type of knowledge stock can be combined to acquire an upgraded production facility. This scheme is shown in Fig. 6, where capabilities are represented by blue squares. In this case capability 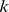 is a combination (blue ellipse) of 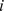 and 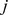. The model capability assumes that if a country has capability 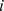 and 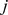, it will acquire 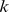 in the next time-step. Each country can in principle acquire the same number of 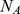 capabilities, endowed with the same set of combination rules. Each capability has on average 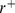 ways by which it may be acquired through combination of two other capabilities (randomly chosen with equal probability).

Each product requires a set of capabilities in order to be produced. In the model a country exports a product if it has *all* necessary capabilities. The simplest possible case (one capability needed for one product) is shown in Fig. 6 where products are represented by red circles. Product 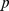 requires capability 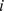, product 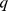 requires 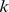. As soon as a country acquires 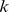, it will start to report exports in 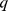. Since one capability may be required for more than one product, all of these products may co-appear with 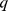. In each country each product requires the same set of 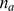 randomly chosen capabilities.

Capabilities can also be lost or abandoned. There is a chance that 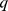 may act as substitute for 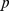. In this case the acquisition of capability 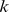 renders 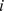 obsolete. With some probability 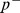 capability 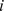 is thus abandoned or destroyed because of 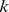. As a consequence 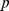 will then no longer be exported. External events are modeled by assuming that in each time step each capability is lost (acquired) with probability 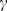 if it was previously existing (not existing).

In the model it is further assumed that the rules of how capabilities can be combined, substituted and used as inputs for products are identical in each country. Economies only differ by their initial diversity of products (from which the implied initial diversity of randomly assigned capabilities is calculated). The model is iterated until the number of model (dis)appearances matches the number of events observed in the trade data. The free model parameters are set to 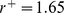, 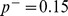, 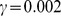, 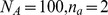. All other model parameters can be measured in the data. The results reported are independent of 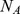 [21], and note that 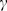 merely sets the time-scale. Fig. 1 shows a comparison of (a) initial and (b) final product diversity, (c) net diversity change and the number of appearances for each (d) country and (e) product for model and trade data. The complex observed patterns of creative destruction can be explained by the almost embarrassingly simple process of recombining and substituting capabilities. The crucial feature of the model is the existence of a creative phase transition with a position depending on the initial diversity [21], [22] and on the parameters 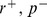. If it is below a certain threshold, there are not enough capabilities available to find successful combinations of them. If a country is at, or above this threshold the creative destruction process kicks in, restructuring the market [2]. This way the model may explain why the whole world is not rich, i.e. the increasing differences in economic development between least developed and developed countries. For a complete model specification, including dynamical algorithm, see the Text S5.

## Discussion

The main novelty of the approach in this article is to identify, in a large sample of countries, the existence of a systematic relationship between the appearance of new industries and the appearance or disappearance of other industries within the same country. In particular our analysis reveals that (i) products appear cluster-wise in creative bursts which consequently (ii) increases the chance for other, already existing products to be pushed from the market and (iii) the emerging products are typically associated with higher income and a higher level of economic complexity. The effect of shifting production towards higher complexity is strongest in the developing countries of Latin America, Eastern Europe and East Asia and Pacific and barely visible in the least developed countries or Europe and North America (see Text S4 and Fig. S4).

These observations can be explained within an evolutionary model of Schumpeterian economic dynamics [2]. Just as the product portfolio of countries can be explained as a consequence of the presence of productive capabilities in that country [8], changes in a country’s product portfolio can be understood through the evolution of its capabilities. Entrepreneurs (firms) upgrade and recombine existing capabilities to create innovations. These may substitute existing industries, restructuring the market. Such a system can exist in three different modes [21], [22]. First, if the initial number of capabilities is too low to allow for a sufficient number of novel combinations, the creative destruction process may not set in at all. Second, if the country is already fully diversified progress is also slowed down. In between these regimes there is a third, transition regime, where the creative destruction process is most effective. We calibrate the model with initial product diversities and (dis)appearance rates observed through world trade data, and can reproduce (i) the position of the transition regime from low to high diversity, (ii) the patterns of (dis)appearance frequencies per country and (iii) the distribution of (dis)appearance frequencies for individual products.

Beyond finding evidence of creative destruction in a large set of national economies, this paper also helps to further reveal the complex topology of industry relatedness. Understanding the drivers of this topology is of the greatest importance for policies to accelerate the economic development of nations.

## Supporting Information

Figure S1
**Appearance and Disappearance events.** Appearance and disappearance events in product *p* in country *c* are only included if the underlying diversity timeseries 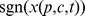 is of one of the types (a)-(d). The time of the appearance event is then always the first appearance (green dashed line in (a) and (b)), disappearances are the last events (red dashed lines in (c) and (d)).(EPS)

Figure S2
**Histograms for Schumpeterian Product Index.** Histograms for Schumpeterian Product Index 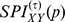 for co-appearances 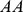, co-disappearances *DD*, and the mixed forms *AD* and *DA* (from top to bottom) for maximal time-lag 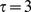. The 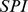 is shown for the surrogate data (blue) and the trade data (red). A significant fraction of higher SPI values for the trade data are seen for appearance-disappearances.(EPS)

Figure S3
**Comparison of PRODY and PCI values for each product category.** The PRODY value was computed as in [19] using NBER trade data as an average over each PRODY from the timespan 1993–2000.To compute the PCI we extract the matrix of significant exporters 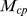 as defined in [7] from the NBER trade data for each year from 1993–2000. Using 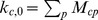 and 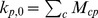 one computes the matrix 
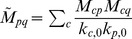
. The PCI is the average over all years of the eigenvector associated with the second largest eigenvalue of 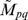. These two indexes are related to each other.(EPS)

Figure S4
**Regional developments in economic complexity.** Histograms for change in (top row) income level 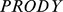 and (bottom row) product complexity 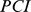 associated with each 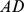 process for seven different world regions (Latin America and the Carribean, East-Asia and the Pacific, Middle-east and North-Africa, Sub-saharan Africa, South-Asia, Western Europe and Northern America, Eastern Europe). There is a skew to the positive side most clearly visible for Latin America and the Carribean, as well as for CEE countries.(EPS)

Figure S5
**Development of economic complexity in Eastern Europe.** Histograms for change in income level 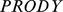 and (a) product complexity 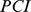 (b) associated with each 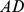 process for Eastern European countries between 1984–1989, that is in a centrally planned economy. The development is more or less symmetric. Compare this to the clear positive skew in (c) and (d), where changes in 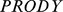 and 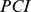 for the same countries between 1990–2000 are shown. This suggests that the increase in economy complexity observed there is due to the shift from planned to market economy.(EPS)

Figure S6 **Number of appearances vs SCI for each country.** This shows that SCI can not be explained by the number of appearances alone. Observe that e.g. Ecuador and Portugal have the same number of product appearances, but Ecuador has one of the highest SCIs and Portugal one of the lowest.
(EPS)

Figure S7 **GDP vs SCI for each country.** Countries cluster into two distinct groups. Western countries are mostly found at the bottom right (low SCI and high GDP), poor countries to the bottom left (low SCI and low GDP), countries with high SCI form a bridge between those two regimes.
(EPS)

Figure S8 **GDP vs average PRODY of products not being exported by this country.** High GDP countries tend to *not* export products with a low PRODY, i.e. low economic complexity (upper left region of the plot). Low GDP countries miss products with a high PRODY in their export baskets, they are found in the lower right region. Oil exporting countries such as Qatar and the United Arabian Emirates deviate from this trend. They achieve high GDP per capita values with significantly less complex products.
(EPS)

Figure S9
**Histograms for Schumpeterian Product Index, excluding CEE.** Histograms for Schumpeterian Product Index 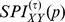 for co-appearances 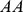, co-disappearances 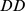, and the mixed forms 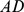 and 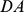 (from top to bottom) for maximal time-lag 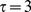. Countries from the Former Soviet Union and Eastern Europe are excluded. The 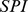 is shown for the surrogate data (blue) and the trade data (red). A significant fraction of higher SPI values for the trade data are seen for appearance-disappearances.(EPS)

Text S1 **A detailed description of the datasets used in this work, as well as the applied filtering and cleaning procedures.**
(PDF)

Text S2 **Further information on the robustness tests for statistical significance of product co-occurrences.**
(PDF)

Text S3 **Discussion of the properties of maximum spanning trees.**
(PDF)

Text S4 **More results and comparisons of different countries and world regions using the indicators discussed in this work.**
(PDF)

Text S5 **The properties of the Schumpeterian evolutionary model are discussed in greater detail, including the description of a dynamical algorithm for simulation purposes.**
(PDF)
