# Supplementary material for: Empirical Confirmation of Creative Destruction from World Trade Data
Source: PLoS One. 2012 Jun 18;7(6):e38924. doi: 10.1371/journal.pone.0038924 (PMC3377723; doi:10.1371/journal.pone.0038924)
Supplement: Text S1 — A detailed description of the datasets used in this work, as well as the applied filtering and cleaning procedures. (PDF) [file pone.0038924.s010.pdf]

1 Description of Datasets

We use two independent data sets in this work.

- **NBER trade data** [1]. Compiled by the National Bureau of Economic Research, this set of bilateral trade data by commodity spans the period 1962-2000. Trade flows (in USD) are reported in product categories following 4-digit SITC rev.2 classification. This dataset is a combination of two others, spanning 1962-1983 and 1984-2000 respectively. We work with the timespan 1984-2000 to exclude any possible artifacts in the results due to changes in data collection between these two timespans. The NBER trade data introduces artificial product categories (containing 'A's and 'X's in the SITC code) to account for differences in import and export records (i.e. if country A exports to countries B,C, but A's export record deviates from (B+C)'s import records). We only focus on export data and exclude these artificial product categories. Finally, we only include 'real' countries (the dataset also lists world regions, such as Southern Asia or Oceania, etc.). This results in longitudinal trade data for 200 countries in 800 product categories over 17 years.

- **COMTRADE trade data** [2]. The United Nations Commodity Trade Statistics Database (UN COMTRADE) publishes annual international trade statistics data by commodities and partner countries. We use data from the timespan 1990-2010. Export values (in USD) are reported in HS1992 product categories for over 170 countries (again, leaving aside world regions), amounting to roughly 5000 categories over 21 years.

Let $A(p,c,t)$ be a product indicator function for the appearance of product p in country c between year $t-1$ and t,

$$A(p,c,t) = \begin{cases} 1 & \text{if } x(p,c,t-1) = 0 \text{ and } x(p,c,t) > 0 \quad , \\ 0 & \text{otherwise} \quad . \end{cases} \tag{1}$$

Similarly the indicator function for a disappearance event is

$$D(p,c,t) = \begin{cases} 1 & \text{if } x(p,c,t-1) > 0 \text{ and } x(p,c,t) = 0 \quad , \\ 0 & \text{otherwise} \quad . \end{cases} \tag{2}$$

Note that these definitions are only useful if there exists a data record for c at both t and $t-1$. We exclude small countries from the analysis by demanding a population of at least 1.2 million people and total exports of at least 1 billion USD, leaving us with a list of 125 countries. The reported results for the SPI where computed over the timespan 1984-2000. Individual trade flows between countries are only included if they exceed 100000 USD Furthermore, appearance and disappearance events are **not** included if one of the following is true.

- In the year before product p appears (or after p disappears) in a country c, the country reports positive exports in less than fifty different categories.

- The diversity time-series $\text{sgn}(x(p,c,t))$ of the product does not belong to one of the four types shown in Fig. S1.

After these filtering procedures we assign each product a appearance or disappearance event (or no event at all). If existing, each product in each country is assigned a unique appearance (disappearance) event which is its first (last) measured event. For this particular event we set $A(p,c,t) = 1$ ($D(p,c,t) = 1$) and zero at each other point, in Fig. S1 appearance events are highlighted by green slashed lines, disappearances by red dashed lines.

2 Result for COMTRADE database

All results reported in the main text can also be found in the COMTRADE database. We have chosen to work with the NBER data for the following reasons. The product classification employed for COMTRADE has gone through several revisions, namely HS1992, HS1996 and HS2002. Each of these revisions causes artificial appearances and disappearances (due to re-classification of products). At the time the dataset was extracted a substantial amount of trade data was still to be reported causing artificial disappearances, thus one can only effectively work with the range 2000-2009. The NBER database, on the other hand, is the outcome a collaborative research effort to provide a coherent dataset. For example, if a country only reports its trade flows in 3-digit SITC or an older revision, this is painstakingly checked against the reports of the trade partners and records from the US trade database to 'fill the gaps'. This reduces the number of artificial appearances and disappearances. A detailed description of how the NBER data was composed can be found in [1]. p-Values for the comparison of trade and surrogate data are shown in Table 1, column 'COMTRADE'

References

1. Feenstra RC, Lipsey RE, Deng H, Ma AC, Mo H (2005) World Trade Flows: 1962-2000. NBER Working Paper Series, 11040. http://www.nber.org/papers/w11040.

2. http://comtrade.un.org/ (retrieved March 2011).
