# Supplementary material for: Empirical Confirmation of Creative Destruction from World Trade Data
Source: PLoS One. 2012 Jun 18;7(6):e38924. doi: 10.1371/journal.pone.0038924 (PMC3377723; doi:10.1371/journal.pone.0038924)
Supplement: Text S2 — Further information on the robustness tests for statistical significance of product co-occurrences. (PDF) [file pone.0038924.s011.pdf]

1 Robustness

Several types of robustness checks are conducted. The analysis is repeated with a threshold of 200,000 USD for export values, that is whenever $x(p,c,t) < 200000$ we set $x(p,c,t) \equiv 0$. The results remain basically unaltered and are thus not driven by small, fluctuating export values, see column 'threshold' in Table 1. The next check concerns artificial product appearances / disappearances due to the creation or abandonment of product categories or countries. To this end only product categories are included in the analysis which have positive world exports in each year. This does not affect the analysis, see column 'all products with positive exports'. Thirdly the influence of the time-span on the results was checked. The time-span was reduced to 1989-2000, see 'cleaned data 1989-2000'. Former communist transition countries from the FSU are excluded in the column 'Excl. FSU'. Then the maximal time lag is varied to $\tau = 5$ and $\tau = 7$ years respectively. Finally the same analysis is carried out with the UN ComTrade data.
