# Supplementary material for: Empirical Confirmation of Creative Destruction from World Trade Data
Source: PLoS One. 2012 Jun 18;7(6):e38924. doi: 10.1371/journal.pone.0038924 (PMC3377723; doi:10.1371/journal.pone.0038924)
Supplement: Text S3 — Discussion of the properties of maximum spanning trees. (PDF) [file pone.0038924.s012.pdf]

1 Maximum Spanning Tree

The MST is the network with the least number of links with the highest possible weights spanning each node of the network. It can be computed using Kruskal's algorithm. The MST can be regarded as the 'skeleton' or 'backbone' of a network. It positions each node in the neighborhood of other nodes to which it has strong ties and is therefore reminiscent of hierarchical clustering procedures.

Networks with an average number of links per node much higher than one are often hard to visualize (usually resulting in something which looks like a 'ball of wool'). This applies even more so to almost fully connected networks, such as the network of co-appearances. In order to arrive at a visually pleasing data representation which allows to directly observe dominating connections in the network it is useful to demand that (i) the minimum number of links spanning the entire set of nodes in the network should be used and simultaneously (ii) that among all possible paths in the network, the one maximizing the link with minimal weight should be chosen. Both conditions are fulfilled by the MST.
