# Supplementary material for: Empirical Confirmation of Creative Destruction from World Trade Data
Source: PLoS One. 2012 Jun 18;7(6):e38924. doi: 10.1371/journal.pone.0038924 (PMC3377723; doi:10.1371/journal.pone.0038924)
Supplement: Text S4 — More results and comparisons of different countries and world regions using the indicators discussed in this work. (PDF) [file pone.0038924.s013.pdf]

1 Further results on country comparisons

Not each region in the world shows the same pattern of development towards higher economic complexity. We repeat the analysis of complexity change associated with AD processes for countries from Latin America and the Carribean, East-Asia and the Pacific, Middle-east and North-Africa, Sub-saharan Africa, South-Asia, Western Europe and Northern America, as well as Eastern Europe separately. That is, we use only countries from the respective regions, compute $SPI_{AD}^{(\tau)}(p)$ for these countries and compute $\Delta PRODY$ and ΔPCI as described in the main text, see Fig. S4. Transitions towards higher economic complexity (skew to the right in the histograms) is clearly visible for Latin America and Eastern Europe. In other regions this trend is less visible or even absent. This supports the notion of 'critical economies' where Schumpeterian economic evolution is successful at work. These critical economies can be identified as transition countries. For low diversity countries (compare e.g. Sub-saharan Africa) there is simply 'too little to start with' in order to move towards higher economic complexity, with respect to this they can be called 'sub-critical'. Fully diversified countries (compare Western Europe and North America) also defy this trend. Since they are already at the top of the 'complexity scale', there is not much room for improvement here. This may be due to an too coarse resolution of the standard product classification. These countries can be dubbed supra-critical.

Eastern European countries have undergone a major shift in their political regimes in the timespan we use for the measurement. One may wonder whether the shift towards higher economic complexity is due to developments under the centrally planned economy before 1990 or afterwards. The analysis is repeated for these countries using the timespan 1984-1989, see Figs. S5(a) and (b). Compare these histograms to the ones observed between 1990-2000 in Figs. S5(c) and (d). There is no clear movement towards higher complexity before 1990. This suggests that the move towards higher complexity observed later is due to the shift from a centrally planned to a free market economy.

In analogy to the SPI a Schumpeterian Country Index SCI is defined for AD and $\tau = 3$ as

$$SCI(c) = \sum_{p,q,t,t'} \left(A(p,c,t)D(q,c,t') - A(q,c,t)D(p,c,t') \right) \quad t < t' \leq t + \tau \quad . \tag{1}$$

We show that the SCI measure is not an artifact of the number of appearances alone in Fig. S6, where the SCI for each country is plotted against the number of appearances in this country. Countries like Portugal and Hongkong have one of the lowest ranking SCIs, Ecuador and Lithuania one of the highest ranking ones. But each of these countries have rougly the same number of product appearances.

Countries with lowest ranking SCI values are countries which are either already fully diversified and show thus only little activity in terms of (dis)appearances (USA, Germany, France, China, UK, ...) or countries with constant low diversities (Malawi, Gabon, Uganda, ...). Countries with a relative high SCI include Uruguay, Kuwait, Croatia, ... and tend to be bridge countries between these two regimes. Fig. S7 shows countries and their SCI and Gross Domestic Product (GDP) per capita of 2000. Western countries are mostly found at the bottom right (low SCI and high GDP), poor countries to the bottom left (low SCI and low GDP), countries with high SCI form a bridge between those two regimes.

The virtues of having or not having a product are shown in Fig. S8. For each country we compute the average PRODY value of products which are *not* exported by this country as of 2000. High GDP countries (compare Canada, Austria, USA, Norway, Sweden, ...) do *not* export products which have on average a significant lower PRODY than developing and least developed countries. There are some outliers, for example oil exporting countries achieving a high GDP per capita with low complexity products.

Since the transition countries from the Former Soviet Union are main contributors to the AD process, as can be observed from the previous results shown in Fig. S4, Fig. S6 and Fig. S7, it is worth to show that the significance of the AD results is not due to the contributions of these countries alone. To this end we compute histograms for $SPI_{XY}^{\tau}(p)$ for a set of countries excluding the FSU and Eastern Europe, see Fig. S9 and the respective column of p-Values in Table 1 entitled 'Excl. FSU'.
