# Supplementary material for: Empirical Confirmation of Creative Destruction from World Trade Data
Source: PLoS One. 2012 Jun 18;7(6):e38924. doi: 10.1371/journal.pone.0038924 (PMC3377723; doi:10.1371/journal.pone.0038924)
Supplement: Text S5 — The properties of the Schumpeterian evolutionary model are discussed in greater detail, including the description of a dynamical algorithm for simulation purposes. (PDF) [file pone.0038924.s014.pdf]

1 Schumpeterian Evolutionary Model

The occurrence of bursts of creation (product appearance) and destruction (product disappearance) is a prominent feature of Schumpeterian economic dynamics [1]. We show that this model can be used to describe the development of national economies in terms of product (dis)appearances as Schumpeterian 'gales of destruction'. The model is a variant of [1] with some features proposed by [2]. The components of the model are now described in detail.

1.0.1 Model Components

Products. For each country c the binary product vector $\pi_{c,p}(t)$ records whether product p is currently produced ($\pi_{c,p}(t) = 1$) or not ($\pi_{c,p}(t) = 0$). To produce p it is required to possess a specific set of capabilities.

Capabilities. A country's product portfolio is determined by the available nontradable capabilities residing in it [2]. Each country c's set of capabilities is described be a time-dependent vector $\sigma_c(t)$ with binary components. The i'th entry in $\sigma_c(t)$ indicates whether the capability i is available in country c at time t ($\sigma_{c,i}(t) = 1$) or is not available ($\sigma_{c,i}(t) = 0$). The total number of capabilities which can be acquired is fixed to some $N_A \gg 1$.

Productions - Capability Gain. Capabilities are acquired through a novel combination of already present capabilities. This is encoded in the production rule table α^+_{ijk}. If capability i can in principle be acquired through a combination of j and k then $\alpha^+_{ijk} = 1$, otherwise $\alpha^+_{ijk} = 0$. Note that each the production rule table is the same for each country. The production process is then given by

$$\sigma_{c,i}(t+1) = \alpha^+_{ijk}\sigma_{c,j}(t)\sigma_{c,k}(t) \quad . \tag{1}$$

There are on average r^+ unique and different production processes for each capability i, with j and k chosen at random from the entire set of capabilities.

Product disappearance. If a product requires a set of capabilities which is a subset of capabilities required to produce another product, it may disappear. The idea is that the product with the larger set of capabilities is a more complex and advanced product substituting its antecedent (e.g. wikipedia replacing an encyclopedia). Assume product p requiring capability i and product q requires capability j. If there is a production rule α^+_{ijk} then product p requires capability j *and* k and is thus an improved version of product q. We incorporate this as competitive replacement in the model by assuming that with probability p^- a production rule for i leads to a negative influence on j. For this the destruction rule table α^-_{ij} is introduced. It encodes whether capability i leads to a substitution for j ($\alpha^-_{ij} = 1$) or not ($\alpha^-_{ij} = 0$). The destruction process is given by

$$\sigma_{c,i}(t+1) = 1 - \alpha^-_{ij}\sigma_{c,j}(t) \quad . \tag{2}$$

The destruction rule table is ubiquitous for each country.

External events. Capabilities can also be lost or acquired through external events. This is modeled by assuming that in each time step each capability is lost (acquired) with probability γ if it was previously existing (not existing).

From Capabilities to Products. Each product needs n_a different capabilities as input. This is accounted for in the capability $\times$ product matrix M_{ip}. If product p requires capability i then $M_{ip} = 1$, otherwise $M_{ip} = 0$. Thus each column has n_a randomly distributed nonzero entries. In each timestep it is checked for each product whether its set of required capabilities is present. Thus $\pi_{c,p}(t) = 1$ if and only if $\sum_i M_{ip}\sigma_{c,i}(t) = n_a$.

1.0.2 Dynamical Algorithm

The Schumpeterian dynamical algorithm for this model is given by:

- Pick a country c at random.
- For each capability i with $\sigma_{c,i}(t) = 1$, set $\sigma_{c,i}(t) = 0$ and $\sigma_{c',i}(t) = 1$ with probability γ (where c' is another randomly chosen country).
- Pick a capability i at random.
- Sum all productive and destructive influences on i, that is compute $\Delta_i(t) \equiv \sum_{j,k} \alpha^+_{ijk}\sigma_{c,j}(t)\sigma_{c,k}(t) - \sum_j \alpha^-_{ij}\sigma_{c,j}(t)$. If $\Delta_i(t) > 0$ ($\Delta_i(t) < 0$) then $\sigma_{c,i}(t+1) = 1$ ($\sigma_{c,i}(t+1) = 0$), otherwise $\sigma_{c,i}(t+1) = \sigma_{c,i}(t)$.
- Continue picking capabilities until all capabilities have been updated, then go to the next country.
- Once all countries have been updated, update the product vectors $\pi_{c,p}(t)$ and go to the next timestep.

The model is initialized with a number of countries N_c and products N_p taken from the data. As further model input serves the number of products that each country exports at the initial time-step $t = 0$ (corresponding to 1984 for the data). Let this diversity be $D_c(0)$. We set $\sigma_{c,i}(0) = 1$ with probability $(D_c(0)/N_p)^{1/n_a}$. This guarantees that the initial model diversity $\sum_p \pi_{c,p}(0)$ equals $D_c(0)$. After initialization, the model is iterated over T' time-steps. Assume that we compare the model to an empirical timeseries over T years. The resulting model trajectories are segmented into T segments of equal length ('model years'). The value of T' is chosen such that the absolute number of appearance and disappearance events is the same in model and real world data. This rescaled model data is now called $\bar{x}(p,c,t)$ and can be compared to the real world data $x(p,c,t)$. We will denote model entities by a bar, e.g. $\bar{A}(p,c,t)$ denotes appearance events obtained from $\bar{x}(p,c,t)$ via the same procedure as for $x(p,c,t)$.

The results of a comparison between the Schumpeterian diversity dynamics model and world trade data are summarized in Fig. 1. Here the model parameter $r^+ = 1.65$, $p^- = 0.15$, $\gamma = 0.002$, $N_A = 100, n_a = 2$. All other model parameters can be measured in the data. In Figs. 1 (a),(b) the initial and final product diversities for each country are shown. In both cases the countries are ranked by diversity. Per calibration the initial diversities are equal. The final diversities (b) also overlap to a large extent. A more detailed view of this is given in Fig. 1 (c). Here the increase in product exports for countries of a given rank is shown. A remarkable peak is visible in both model and trade data. In the model this is explained by the onset of a creative phase transition [3], [4]. As a country gradually acquires novel capabilities it eventually reaches a point where it has accumulated enough critical capabilities to diffuse into the entire product space. Creative bursts of diversification are the consequence. Countries below this critical point experience stagnation, whereas countries above this threshold reach a fully diversified plateau.

The results reported are independent of N_A [3]. The variable γ merely sets the time-scale for T'. The parameters r^+ and p^- determine the number of capabilities required for creative bursts to set in, that is the phase transition to a fully diversified country. Changing these values rescales the peak in Fig. 1 (c). The main conclusions however remain intact.

References

1. Thurner S, Klimek P, Hanel R (2010) Schumpeterian Economic Dynamics as a Quantifiable Minimum Model of Evolution. *New J Phys* 12: 075029.

2. Hidalgo CA, Hausmann R (2009) The Building Blocks of Economic Complexity. *Proc Natl Acad Sci USA* 106 (26): 10570-5.

3. Hanel R, Thurner S, Kauffman SA (2005) The Phase Transition of Random Catalytic Sets. *Phys Rev E* 72: 036117.

4. Hanel R, Thurner S, Kauffman SA (2007) Towards a physics of evolution: critical diversity dynamics at the edges of collapse and bursts of diversification. *Phys Rev E* 76: 036110.
